# Supplementary material for: Recurrence Patterns and Risk Factors after Curative Resection for Colorectal Cancer: Insights for Postoperative Surveillance Strategies
Source: Cancers (Basel). 2023 Dec 10;15(24):5791. doi: 10.3390/cancers15245791 (PMC10742009; doi:10.3390/cancers15245791)
Supplement: Supplementary file 1 [file cancers-15-05791-s001.zip › cancers-2684896-supplementary.pdf]

Table S1 Clinical and tumor characteristics of colon cancer patients according to recurrence

	Recurrence (n=141)	No Recurrence (n=1450)	*P* value
Age, years (Mean ± SD)	64.8 ± 13.0	64.2 ± 11.7	0.58
Sex, n (%)			0.95
Male	81 (57.4)	837 (57.7)	
Female	60 (42.6)	613 (42.3)	
Body mass index, kg/m^2 (Mean ± SD)	23.6 ± 3.0	23.8 ± 3.3	0.43
ASA score, n (%)			0.77
1	29 (20.6)	267 (18.4)	
2	96 (68.1)	1059 (73.0)	
3	14 (9.9)	116 (8.0)	
4	2 (1.4)	8 (0.6)	
Comorbidities, n (%)			
Endocrine	25 (17.7)	317 (21.9)	0.26
Cardiovascular	69 (48.9)	695 (47.9)	0.82
Respiratory	16 (11.3)	122 (8.4)	0.24
Smoking, n (%)	46 (32.6)	421 (29.0)	0.39
Alcohol, n (%)	65 (46.1)	577 (39.8)	0.15
Tumor location, n (%)			0.096
Ascending colon	44 (31.2)	366 (25.2)	
Transverse colon	23 (16.3)	238 (16.4)	
Descending colon	10 (7.1)	75 (5.2)	
Sigmoid colon	64 (45.4)	771 (53.2)	
CEA level ≥ 5 ng/mL, n (%)	38 (27.0)	283 (19.5)	0.016
Obstruction, n (%)	44 (31.2)	155 (10.7)	<0.001
Perforation, n (%)	5 (3.5)	43 (3.0)	0.72
Endoscopic stent insertion, n (%)	20 (14.2)	63 (4.3)	<0.001
Adjuvant chemotherapy, n (%)	93 (66.0)	677 (46.7)	<0.001

ASA, American Society of Anesthesiologists physical status classification system; CEA, Carcinoembryonic antigen; SD, Standard deviation

Table S2 Surgical and pathological characteristics of colon cancer patients according to recurrence

	Recurrence (n=141)	No Recurrence (n=1450)	*P* value
Emergency operation, n (%)	8 (5.7)	35 (2.4)	0.046
Open conversion, n (%)	21 (14.9)	77 (5.3)	0.002
R2 resection, n (%)	2 (1.4)	4 (0.3)	0.087
Estimated blood loss ≥ 800ml	8 (5.7)	22 (1.5)	<0.001
Operation time ≥ 240 min	30 (21.3)	194 (13.4)	<0.001
Transfusion, n (%)	17 (12.1)	77 (5.3)	<0.001
Anastomotic leakage, n (%)	3 (2.1)		0.002
Pathologic T stage, n (%)			<0.001
T1	2 (1.4)	232 (16.0)	
T2	7 (5.0)	205 (14.1)	
T3	100 (70.9)	908 (62.6)	
T4	32 (22.7)	105 (7.2)	
Pathologic N stage, n (%)			<0.001
N0	56 (39.7)	943 (65.0)	
N1	53 (37.6)	389 (26.8)	
N2	32 (22.7)	118 (8.1)	
TNM Stage, n (%)			<0.001
Ⅰ	5 (3.5)	361 (24.9)	
Ⅱ	51 (36.2)	582 (40.1)	
Ⅲ	85 (60.3)	507 (35.0)	
Harvested LN < 12, n (%)	8 (5.7)	138 (9.5)	0.17
Differentiation, n (%)			0.095
Well differentiated	28 (19.9)	274 (18.9)	
Moderate differentiated	97 (68.8)	1071 (73.9)	
Poorly differentiated	7 (5.0)	38 (2.6)	

Mucinous	7 (5.0)	63 (4.3)	
Signet ring cell	2 (1.4)	1 (0.1)	
Etc.	0	3 (0.2)	
Lymphatic invasion, n (%)	41 (29.1)	234 (16.1)	0.06
Vascular invasion, n (%)	18 (12.8)	37 (2.6)	0.039
Perineural invasion, n (%)	16 (11.3)	43 (3.0)	<0.001
DRM involvement, n (%)	0	1 (0.1)	0.017
CRM involvement, n (%)	1 (0.7)	14 (1.0)	0.039

LN, Lymph node; DRM, Distal resection margin; CRM, Circumferential resection margin

Table S3 Clinical and tumor characteristics of rectal cancer patients according to recurrence

	Recurrence (n=150)	No Recurrence (n=881)	*P* value
Age, years (Mean ± SD)	61 ± 12.6	61.6 ± 12.0	0.63
Sex, n (%)			0.033
Male	103 (68.7)	576 (65.4)	
Female	47 (31.3)	305 (34.6)	
Body mass index, kg/m^2 (Mean ± SD)	23.4 ± 3.5	23.6 ± 3.1	
ASA score, n (%)			
1	41 (27.3)	199 (22.6)	
2	99 (66.0)	634 (72.0)	
3	10 (6.7)	48 (5.4)	
4	0	0	
Comorbidities, n (%)			
Endocrine	36 (24.0)	169 (19.2)	0.17
Cardiovascular	61 (40.7)	358 (40.6)	0.99
Respiratory	11 (7.3)	64 (7.3)	0.98
Smoking, n (%)	54 (36.0)	314 (35.6)	0.93
Alcohol, n (%)	55 (36.7)	364 (41.3)	0.28
Tumor location, n (%)			0.082
Upper rectum	15 (10.0)	136 (15.4)	

Mid/Low rectum	135 (90.0)	745 (84.6)	
CEA level ≥ 5 ng/mL, n (%)	38 (25.3)	151 (17.1)	0.023
Obstruction, n (%)	6 (4.0)	26 (3.0)	0.54
Perforation, n (%)	3 (2.0)	4 (0.5)	0.033
Endoscopic stent insertion, n (%)	4 (2.7)	9 (1.0)	0.095
Neoadjuvant Therapy, n (%)	66 (44.0)	245 (27.8)	<0.001
SCRT	9 (6.0)	40 (4.5)	
LCRT	57 (38.0)	205 (23.3)	
Adjuvant chemotherapy, n (%)	105 (70.0)	441 (50.1)	<0.001
Postoperative radiotherapy, n (%)	5 (3.3)	26 (3.0)	0.81

ASA, American Society of Anesthesiologists physical status classification system; CEA, Carcinoembryonic antigen; SD, Standard deviation; SCRT, Short-course radiotherapy; LCRT, Long-course radiotherapy

Table S4 Surgical and pathological characteristics of rectal cancer patients according to recurrence

	Recurrence (n=150)	No Recurrence (n=881)	*P* value
Emergency operation, n (%)	1 (0.7)	0	0.015
Open conversion, n (%)	2 (1.3)	15 (1.7)	0.72
R2 resection, n (%)	1 (0.7)	4 (0.5)	0.76
Estimated blood loss ≥ 800ml	7 (4.7)	13 (1.5)	0.009
Operation time ≥ 240 min	94 (62.7)	403 (45.7)	<0.001
Transfusion, n (%)	18 (12.0)	44 (5.0)	0.001
IMA high ligation, n (%)	125 (83.4)	707 (80.3)	0.63
Lateral pelvic LN dissection, n (%)	7 (4.7)	26 (3.0)	0.27
Anastomotic leakage, n (%)	26 (17.3)	79 (9.0)	0.002
Pathologic T stage, n (%)			<0.001

T0	2 (1.3)	22 (2.5)	
T1	4 (2.7)	125 (14.2)	
T2	23 (15.3)	230 (26.1)	
T3	104 (69.3)	488 (55.4)	
T4	17 (11.3)	16 (1.8)	
Pathologic N stage, n (%)			<0.001
N0	64 (42.7)	593 (67.3)	
N1	47 (31.3)	207 (23.5)	
N2	39 (26.0)	81 (9.2)	
Lateral pelvic LN metastasis, n (%)	0	4 (0.5)	0.045
TNM Stage, n (%)			<0.001
0	2 (1.3)	18 (2.0)	
Ⅰ	19 (12.7)	288 (32.7)	
Ⅱ	43 (28.7)	287 (32.6)	
Ⅲ	86 (57.3)	288 (32.7)	
Harvested LN < 12, n (%)	34 (22.7)	134 (15.2)	0.022
Differentiation, n (%)			0.35
Well differentiated	35 (23.3)	159 (18.0)	
Moderate differentiated	105 (70.0)	679 (77.1)	
Poorly differentiated	6 (4.0)	10 (1.1)	
Mucinous	4 (2.7)	30 (3.4)	
Signet ring cell	0	2 (0.2)	
Etc.	0	1 (0.1)	
Lymphatic invasion, n (%)	23 (15.3)	114 (12.9)	0.92
Vascular invasion, n (%)	9 (6.0)	30 (3.4)	0.95
Perineural invasion, n (%)	19 (12.7)	35 (4.0)	0.005
DRM involvement, n (%)	3 (2.0)	4 (0.5)	0.033
CRM involvement, n (%)	8 (5.3)	20 (2.3)	0.033

IMA, Inferior mesenteric artery; LN, Lymph node; DRM, Distal resection margin; CRM, Circumferential resection margin

Table S5 The distribution of neoadjuvant and adjuvant therapies according to stages of colon and rectal cancer patients

	[a] Neoadjuvant treatment		Adjuvant chemotherapy	
	Colon cancer	Rectal cancer	Colon cancer	Rectal cancer
Stage 0, n (%)				2/20 (10.0)
Stage Ⅰ, n (%)	0/395	16/174 (9.2)	11/366 (3.0)	14/307 (4.6)
Stage Ⅱ, n (%)	0/432	23/95 (24.2)	255/366 (69.7)	209/330 (63.3)
Stage Ⅲ, n (%)	9/764 (1.2)	272/762 (35.7)	504/592 (85.1)	321/374 (85.8)

[a] The stage for neoadjuvant treatment is the clinical stage.

Table S6 Univariate and Multivariate analyses of risk factors associated with recurrence in colon cancer patients (N=1591)

	Univariate analysis		Multivariate analysis	
**Variables**	HR (95% CI)	*P*	HR (95% CI)	*P*
**Patient Factor**				
Age	1.01 (0.99-1.02)	0.37		
Sex (F)	0.99 (0.72-1.39)	0.99		
BMI	0.97 (0.93-1.03)	0.32		
ASA status (1/2 vs. 3/4)	1.46 (0.87-2.45)	0.16		
Comorbidities				
Endocrine	0.78 (0.51-1.20)	0.26		
Cardiovascular	1.05 (0.76-1.46)	0.77		
Respiratory	1.42 (0.84-2.38)	0.19		
Alcohol	1.20 (0.85-1.71)	0.30		
Smoking	1.27 (0.91-1.77)	0.16		
**Clinical factor**				
**Obstruction**	**3.50 (2.45-5.00)**	**<0.001**	**2.57 (1.53-4.31)**	**<0.001**
Perforation	1.28 (0.53-3.14)	0.58		
CEA≥5	1.63 (1.11-2.38)	0.013	1.02 (0.68-1.55)	0.94
**Surgical factor**				
Emergency operation	2.48 (1.22-5.06)	0.013	0.73 (0.25-2.12)	0.57
Open conversion	3.03 (1.90-4.81)	<0.001	1.41 (0.57-3.48)	0.45
Estimated blood loss ≥ 800ml	3.70 (1.81-7.56)	<0.001	1.13 (0.42-3.04)	0.81
Operation time ≥ 240 min	1.69 (1.13-2.53)	0.011	1.37 (0.86-2.17)	0.18
Transfusion	2.39 (1.44-3.97)	0.001	1.77 (0.90-3.49)	0.10
Anastomosis leakage	0.77 (0.24-2.40)	0.65		
LN < 12	0.58 (0.28-1.18)	0.13		
**Tumor factor**				
Location (Rt vs. Lt)	0.80 (0.58-1.12)	0.19		

TNM stage				
**T1/T2 vs. pT3/T4**	**5.89 (3.00-11.58)**	**<0.001**	**3.42 (1.61-7.26)**	**0.001**
**N0 vs N1/N2**	**2.69 (1.92-3.77)**	**<0.001**	**2.45 (1.58-3.80)**	**<0.001**
Differentiation (WD/MD vs. PD/MUC/SRC)	1.60 (0.95-2.69)	0.076		
**Vascular invasion**	**4.71 (2.87-7.72)**	**<0.001**	**3.07 (1.65-5.70)**	**<0.001**
Lymphatic invasion	2.10 (1.46-3.02)	<0.001	1.09 (0.70-1.69)	0.69
Perineural invasion	3.98 (2.36-6.70)	<0.001	1.47 (0.76-2.82)	0.25
DRM		0.84		
CRM	0.84 (0.12-5.99)	0.86		
**Treatment factor**				
Stent insertion	3.31 (2.06-5.31)	<0.001	0.72 (0.26-1.96)	0.51
Adjuvant chemotherapy	1.94 (1.37-2.74)	<0.001	0.67 (0.43-1.06)	0.085

Table S7 Univariate and Multivariate analyses of risk factors associated with recurrence in rectal cancer patients (N=1031)

	Univariate analysis		Multivariate analysis	
**Variables**	HR (95% CI)	*P*	HR (95% CI)	*P*
**Patient Factor**				
Age	0.999 (0.98-1.01)	0.84		
Sex (M)	1.15 (0.82-1.63)	0.42		
BMI	0.97 (0.92-1.02)	0.28		
ASA status (1/2 vs. 3/4)	1.37 (0.72-2.61)	0.34		
Comorbidities				
Endocrine	1.36 (0.93-1.97)	0.11		
Cardiovascular	1.03 (0.74-1.42)	0.88		
Respiratory	1.06 (0.57-1.95)	0.86		
Alcohol	0.83 (0.60-1.16)	0.28		
Smoking	1.02 (0.73-1.43)	0.90		
**Clinical factor**				
Obstruction	1.50 (0.66-3.40)	0.33		
**Perforation**	**3.67 (1.17-11.52)**	**0.026**	**4.38 (1.34-14.34)**	**0.015**
CEA≥5	1.68 (1.16-2.44)	0.006	1.30 (0.88-1.91)	0.19
**Surgical factor**				
**Emergency operation**	**30.85 (4.22-225.77)**	**0.001**	**43.95 (5.70-338.96)**	**<0.001**
Open conversion	0.82 (0.20-3.29)	0.77		
IMA high vs. low ligation	1.07 (0.46-2.47)	0.88		
LPLND	0.75 (0.82-3.75)	0.15		
**Estimated blood**	**3.27 (153-6.98)**	**0.002**	**2.76 (1.17-6.54)**	**0.021**

**loss ≥ 800ml**				
Operation time ≥ 240 min	1.85 (1.33-2.58)	<0.001	1.67 (0.95-1.96)	0.09
Transfusion	2.37 (1.45-3.89)	0.001	1.51 (0.86-2.64)	0.15
**Anastomosis leakage**	**2.14 (1.40-3.27)**	**<0.001**	**2.01 (1.29-3.12)**	**0.002**
**LN < 12**	**1.50 (1.02-2.20)**	**0.038**	**1.69 (1.12-2.54)**	**0.013**
**Tumor factor**				
Location (UR vs. MR/LR)	1.55 (0.91-2.64)	0.11		
TNM stage				
**T1/T2 vs. pT3/T4**	**2.98 (1.98-4.46)**	**<0.001**	**2.19 (1.35-3.55)**	**0.001**
**N0 vs N1/N2**	**2.67 (1.93-3.68)**	**<0.001**	**2.53 (1.69-3.79)**	**<0.001**
LPLN metastasis	0.05 (0.00-1838.36	0.58		
Differentiation (WD/MD vs. PD/MUC/SRC)	1.48 (0.78-2.81)	0.23		
Vascular invasion	2.15 (1.09-4.21)	0.026	0.94 (0.42-2.11)	0.88
Lymphatic invasion	1.24 (0.80-1.94)	0.34		
Perineural invasion	3.38 (2.09-5.48)	<0.001	1.75 (0.99-3.08)	0.055
**DRM**	**5.36 (1.70-16.83)**	**0.004**	**4.15 (1.17-14.79)**	**0.028**
**CRM**	**2.44 (1.20-4.97)**	**0.014**	**2.18 (1.04-4.54)**	**0.038**
**Treatment factor**				
**Stent insertion**	**2.77 (1.03-7.49)**	**0.044**	**2.90 (1.05-8.01)**	**0.041**
**Neoadjuvant Tx**	**1.96 (1.42-2.71)**	**<0.001**	**1.70 (1.18-2.44)**	**0.005**
SCRT	1.89 (0.95-3.76)	0.071		
LCRT	2.03 (1.44-2.84)	<0.001		
Adjuvant chemotherapy	1.97 (1.39-2.80)	<0.001	0.76 (0.49-1.20)	0.25
Postoperative RT	1.11 (0.46-2.72)	0.81		

CEA, Carcinoembryonic antigen; LN, Lymph node; DRM, Distal resection margin; CRM, Circumferential resection margin.
